# Technical feasibility and safety profile of laparoscopic diverted sleeve gastrectomy with ileal transposition (DSIT)

**DOI:** 10.1007/s11695-014-1518-1

**Published:** 2014-12-02

**Authors:** Alper Celik, Surendra Ugale, Hasan Ofluoglu, Muharrem Asci, Bahri Onur Celik, Erol Vural, Mustafa Aydin

**Affiliations:** 1 Metabolic Surgery Clinic, Taksim German Hospital, Istanbul, Turkey; 2 Department of General Surgery, Medical Faculty, Yeniyuzyil University, Istanbul, Turkey; 3 Kirloskar Hospital, Hyderabad, India

**Keywords:** Laparoscopic diverted sleeve gastrectomy, Ileal transposition, Type 2 diabetes

## Abstract

**Background:**

In this study, we specifically aimed to analyze the technical and safety aspects of laparoscopic diverted sleeve gastrectomy with ileal transposition (DSIT) in patients with type 2 diabetes (T2DM).

**Methods:**

A total of 360 patients with type 2 diabetes who underwent a DSIT procedure within the past 2-year period (2011–2013) were analyzed. Operation time, length of hospital stay, perioperative and postoperative complications, and mortality were recorded and analyzed.

**Results:**

The participants consisted of 229 males (63.6 %) and 131 females (36.4 %). Mean duration of follow-up was 12.4 months (range, 6–31). One early and two late mortalities occurred. Early mortality was due to an anastomotic leak, and late mortalities were related to myocardial infarction and a traffic accident. Leakage and bleeding were the most common surgical complications. The total number of surgical complications was 22 (6.1 %). Of those, 19 occurred within the first month (early) and 3 occurred after the first month (late). Among early complications, seven (1.94 %) required re-operation, two patients (0.55 %) required endoscopic treatment, and the remaining ten were managed conservatively. Late surgical complications (sleeve angulation) were noted in three patients (0.83) and were treated by endoscopic stents. Surgery-related non-surgical complications occurred in 19 cases (5.2 %), and neurological complications were noted in 11 patients (3.05 %). Additional surgical intervention was required in 26 patients (7.22 %). Of those, 15 (4.16 %) required cholecystectomy.

**Conclusions:**

Our data demonstrate that DSIT is a technically feasible operation and can be safely performed in type 2 diabetic patients with acceptable complication and mortality rates.

## Introduction

The continuous increase in the prevalence of obesity and the associated type 2 diabetes in the last two decades have evidently reached the scale of a global epidemic. Thus, it is not surprising to observe that currently, type 2 diabetes represents the fifth leading cause of death in the USA [1]. Despite escalating efforts to develop novel and effective medical and/or behavioral therapeutic options for type 2 diabetes, at present, only surgery can offer highest success rates with sustainable results [2, 3]. However, this does not mean that the ultimate surgical approach has been devised and standard surgical techniques have been defined. On the contrary, a variety of restrictive, malabsorptive, and/or combined techniques have become a part of the growing trend of metabolic and bariatric surgery and are increasingly more frequently used for a wider range of body mass index (BMI) values than before for the treatment of the dynamic, heterogeneous, and multifactorial condition. Majority of the common techniques used in obese diabetics have focused on easier and simpler methods, consisting of either restriction or combination of restriction with exclusion of the foregut. Malabsorptive techniques have focused primarily on the hindgut stimulation. This results in a great variation in the reported functional success rates, additional drug requirements, and the need for revisions.

The risk of morbidity, mortality, and in particular digestive or metabolic complications associated with surgery should also be taken into consideration. Furthermore, several effects commonly seen after surgery such as the change in eating habits, nausea, and reflux should be carefully addressed, especially in terms of their effects on the quality of life. Also, increased toilet regularity after malabsorptive surgeries can seriously affect a patient’s social life [4].

Metabolic surgery is an evolving field of medicine where novel surgical techniques are continuously being searched for an acceptably sustainable metabolic outcome with minimal mortality, perioperative morbidity, and lower risk of long-term complications. An ideal or near-ideal procedure should also be able to minimize the alterations in nutrition and quality of ingestion and provide simple preventive and therapeutic strategies for the non-morbidly obese diabetics who already have a lower nutritional reserve compared to the morbidly obese patients.

The efficacy and safety of laparoscopic diverted sleeve gastrectomy with ileal transposition in diabetic patients with lower BMIs have been previously shown [5, 6]. We hereby document the outcomes in a wider range of patients and report on mortality and mortality, with a special emphasis on non-surgical complications.

## Materials and Methods

Institutional ethical committee approval was obtained before the study was commenced. In this report, we have retrospectively analyzed our prospectively collected data on type 2 diabetic patients treated with laparoscopic diverted sleeve gastrectomy with ileal transposition (DSIT) in a single “Center of Excellence in Bariatric and Metabolic Surgery”, which is a certified institution. A total of 454 patients were operated under the same clinical and laboratory protocol between October 2011 and December 2013 by the same surgical team.

Inclusion criteria were as follows: a ≥3-year history of type 2 diabetes (T2DM) under stable medical treatment, HbA1c >7 % for more than 3 months, weight stability defined as no significant change (>3 %) of weight within the past 3 months, and BMI above 25 kg/m^2^. Patients were excluded if they had a BMI below 25 kg/m^2^, fasting C peptide level below 0.5 ng/ml, or antiGAD antibody positivity. Other exclusion criteria included previous major gastrointestinal surgery, pregnancy, and inability to tolerate anesthesia. Patients with severe eating problems and those on medications for eating disorders were also excluded. A total of 360 patients followed up for 1 year were finally included in the study.

**Fig. 1:**
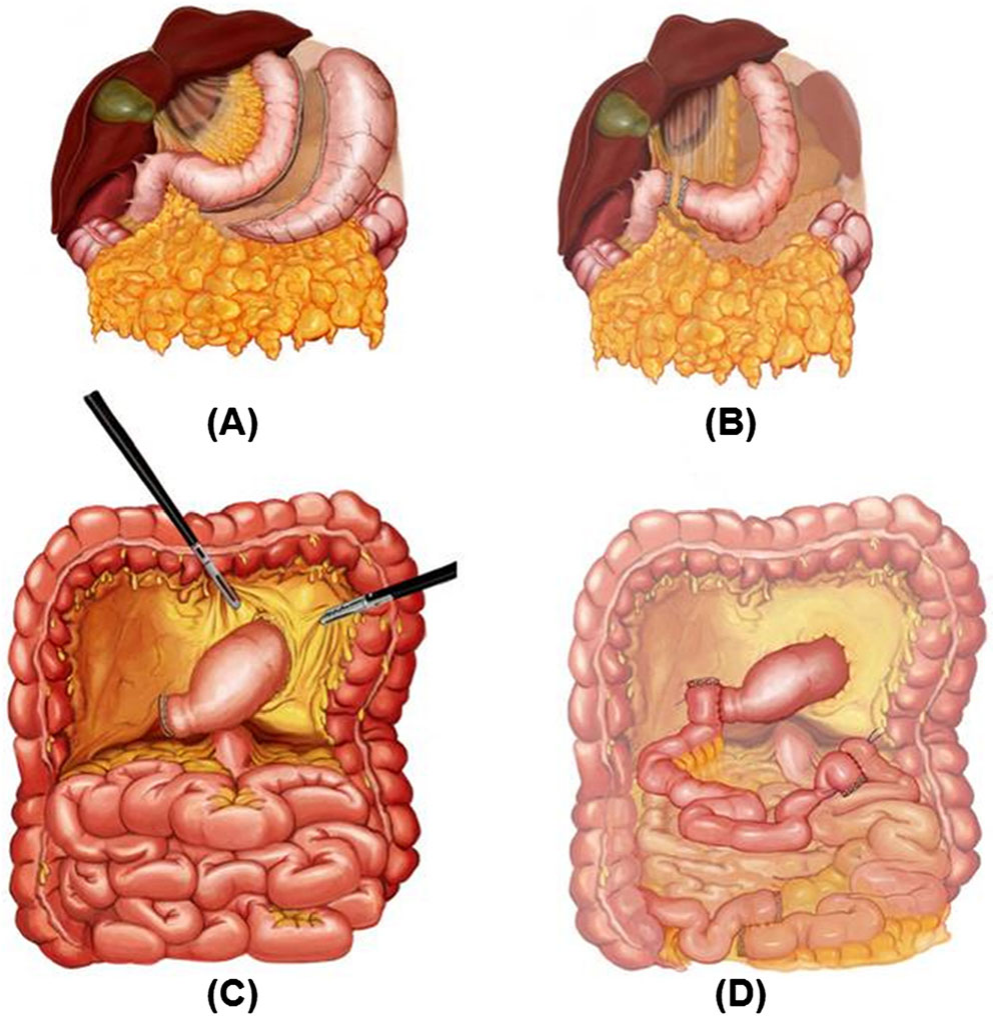
Schematic demonstration of the operation. **a** Sleeve gastrectomy. **b** Duodenal transection. **c** Inframesocolic transfer of the sleeve. **d** Interposition of the ileal segment between distal stomach and the proximal jejunum

The overall study population consisted of 360 poorly controlled (mean HbA1c 9.41 %) diabetics (229 male, 63.6 %; 131 female, 36.4 %) with a mean age of 51.2 years (range, 24–79 years) and diabetes duration of 12.6 years (range, 3–31 years). All patients were evaluated preoperatively by an extended 3-day routine checkup, 1 month prior to surgery. This evaluation included routine blood analysis and routine electromyography (EMG), Doppler ultrasound examination of the carotid arteries and lower extremities, echocardiography (ECHO), and computed tomography angiography (CT-Angio) of the coronary vessels. In total, there were 71 (19.7 %) previous cardiovascular events. All cardiovascular events are listed in Table 1. All patients underwent the same operation (laparoscopic diverted sleeve gastrectomy with ileal transposition, (DSIT)) performed by the same team as previously described [7]. Briefly, in this procedure, we start with a sleeve gastrectomy or fundectomy (depending on the BMI) and progress with duodenal transection 2–3 cm from the pylorus. The sleeved stomach is transferred to the lower abdomen through a transverse meso-colic opening. A single stay suture is placed 50 cm from the ligament of Treitz, and the cecum is identified. The last 30 cm of ileum is preserved, and a 170-cm segment of the distal ileal segment is prepared for the anastomosis. The first anastomosis is ileo-ileostomy, the second is duodeno-ileostomy, and the third is ileo-jejunostomy. The first and the last anastomoses are done in a functional side-to-side manner, and the second anastomosis is done hand sewn with a single-layer continuous 3/0 polydiaxanone (PDS) suture. All the mesenteric defects are closed using 3/0 polypropylene sutures. Schematic demonstration of the operation is shown in Fig. 1. All patients operated after 2012 underwent routine synchronous cholecystectomy. Eight patients underwent additional Meckel’s diverticulectomy at the time of surgery. None of them developed any postoperative complications. A detailed record and analysis of all procedures, including duration of surgery, perioperative, and postoperative adverse events, were maintained. All patients received routine multivitamin supplements for at least 6 months after surgery.

**Table 1:** Previous CVE events in our patients who underwent DSIT

Cardiovascular event	*N* (%)
Atrial fibrillation	4 (1.1)
Compensated congestive cardiac failure	4 (1.1)
Moderate valvulopathy	4 (1.1)
Carotid stenosis	22 (6.1)
Previous myocardial infarction	5 (1.38)
Previous CABG	12 (3.3)
Previous coronary stent	18 (5)
Previous carotid surgery	2 (0.55)
Total	71 (19.7)

It should be noted that around 20 % of patients had a major cardiac or vascular problem before surgery, demonstrating the heavy vascular burden of our patient profile

## Results

A total of 360 patients followed up for 1 year were finally included in the study. All patients had a BMI above 25 kg/m^2^. Nearly one third of the patients (34.3 %) had a BMI above 35, and two thirds (65.7 %) had a BMI below 35 kg/m^2^. Majority of the patients (42 %) had a BMI between 30 and 35 kg/m^2^. Patient distribution according to BMI and the percentage of BMI loss (BMIL%) are shown in Table 2.

**Table 2:** Changes in BMI were analyzed using four BMI subcategories: BMI <30 kg/m^2^, BMI = 30–34.9 kg/m^2^, BMI = 35–39.9 kg/m^2^, and BMI > 40 kg/m^2^. Increasing BMI was associated with a tendency towards more weight loss, as expected

	BMI <29.9	BMI = 30–34.9	BMI = 35–39.9	BMI > 40
Mean preoperative BMI	27.9 kg/m^2^	32.1 kg/m^2^	37.2 kg/m^2^	43 kg/m^2^
Mean postoperative BMI	21.63 kg/m^2^	24.19 kg/m^2^	26.79 kg/m^2^	29.8 kg/m^2^
Change in mean BMI	6.27 kg/m^2^	7.91 kg/m^2^	10.41 kg/m^2^	13.2 kg/m^2^
Percentage change in BMI (BMIL%)	22.4 %	24.64 %	27.98 %	30.3 %

Improvement/remission in T2DM and in comorbidities will be reported separately in another publication. Comorbid conditions included hypertension in 226 patients (62.7 %), hypercholesterolemia in 217 (60.27 %), and hypertriglyceridemia in 170 subjects (47.2 %). End-organ damage was noted in 84 patients (23.3 %), with 8 patients (2.2 %) having nephropathy, 37 (10.27 %) retinopathy, and 39 (10.83 %) severe, EMG proven neuropathy.

The outcomes are summarized in 5 categories: mortality (A), surgical complications (B), surgery-related non-surgical complications (C), neurological complications (D), and patients requiring additional surgery (E). Complications such as diarrhea, excessive weight loss, reflux, and itching will be discussed within the topic of surgery-related non-surgical complications (B).

(A) Mortality: There were three mortalities. One patient died 20 days after surgery due to ileo-jejunal anastomotic leak. One patient in complete remission died 8 months after surgery due to myocardial infarction, and another patient in complete remission died 13 months after surgery in a traffic accident (he was not the driver). Surgery-related mortality rate was 0.27 %.
(B) Surgical complications (*N*, 22; 6.1 %): In total, there were 22 surgical complications (6.1 %). Of these, 8 (2.2 %) were leaks (4 anastomotic, 2 bile leaks, 2 duodenal stump leaks); 5 had bleeding (3 intra-abdominal, 2 intraluminal); 3 strictures (2 duodeno-ileal anastomotic, 1 biliary stricture); 3 sleeve angulations; 1 abscess; 1 wound infection; and 1 deep vein thrombosis. Among the 22 patients with surgical complications, 7 patients required immediate surgical re-intervention (1.94 %). The remaining 15 were managed conservatively. There was no sleeve leak and no mesenteric herniation. Table 3 shows the nature and the number of the surgical complications.

**Table 3:** A total of 22 surgical complications occurred among 360 patients undergoing DSIT

Surgical complications: *n*, 22 (6.1 %)				
Leak: *n*, 8 (2.2 %)	Bleeding: *n*, 5 (1.38 %)	Infectious: *n*, 2 (0.55 %)	Stricture/angulation: *n*, 6 (1.38 %)	Other: *n*, 1 (0.27 %)
Anastomotic leak: 4 (1.1 %)	Intra-abdominal: 3 (0.83 %)	Abdominal abscess: 1 (0.27 %)	Anastomotic: 2 (0.55 %)	DVT: 1 (0.27 %)
Duodenal stump leak: 2 (0.55 %)	Intraluminal: 2 (0.55 %)	Wound infection: 1 (0.27 %)	Biliary stricture: 1 (0.27 %)	
Bile leak: 2 (0.55 %)			Sleeve angulation: 3 (0.83 %)	

The most frequent complication was leak (2.2 %), followed by bleeding (1.38 %), stricture or sleeve angulation (1.38 %), and DVT (0.27 %). Abdominal abscess occurred in the second patient with duodenal stump leak, and the patient developed abscess despite adequate drainage and antibiotic coverage. Bile leaks resulted from misplaced cystic duct clips in one case and a Luschka leak in the second. There were no sleeve leaks and no mesenteric hernia
(C) Surgery-related non-surgical complications (*N*, 20; 5.5 %): We observed surgery-related non-surgical complications in 20 cases. The most frequent complaint was the change in bowel habits, reported by ten patients (2.77 %). Diarrhea occurred in seven patients and constipation in three. We observed food intolerance (without a mechanical reason) occurring in five, excessive weight loss (without nausea and vomiting) in two, intractable reflux in one, hypoglycemia in one, and itching in one case. Surgery-related non-surgical complications are shown in Table 4.

**Table 4:** The most common non-surgical complaint was a change in bowel movements that occurred in 10 subjects

Surgery-related non-surgical complications: *n*, 20 (5.5 %)
Change in bowel movements: *n*, 10 (2.77 %)
Food intolerance (without a mechanical reason): *n*, 5 (1.38 %)
Excessive weight loss (without nausea and vomiting): *n*, 2 (0.55 %)
Intractable reflux: *n*, 1 (0.27 %)
Itching: *n*, 1 (0.27 %)
Hypoglycemia: *n*, 1 (0.27 %)

Among them, seven had diarrhea and three had constipation. In total, two cases had a BMI below 20 kg/m^2^ 4 and 5 months after surgery, respectively. These patients were hospitalized and received appropriate treatment. Six months after surgery, they both reached a BMI above 20 kg/m^2^. Other rare complications were reflux, itching, and hypoglycemia
(D) Neurological complications (*N*, 11; 3.05 %): Totally, 11 patients had neurological complications. Two patients complained worsening of amnesia and another three complained worsening of polyneuropathy (PNP). Three patients experienced a unilateral foot drop (peroneal palsy) that responded to medical treatment, and another three patients had a cerebrovascular event. None of them were fatal. Neurological complications are shown in Table 5.

**Table 5:** The total rate of neurological complications was approximately 3 %

Neurological complications: *n*, 11 (3.05 %)
Cerebrovascular event: *n*, 3 (0.83 %)
Foot drop (peroneal palsy): *n*, 3 (0.83 %)
Worsening of polyneuropathy: *n*, 3 (0.83 %)
Worsening of amnesia: *n*, 2 (0.55 %)

Of note, two patients developed a hemorrhagic and one patient developed an ischemic cerebrovascular event. None of them were fatal. All cases responded to medical treatment and physiotherapy
(E) Additional surgical intervention (*N*, 26; 7.22 %): In total, 26 patients required an additional surgical procedure after the DSIT. Of those, 15 were cholecystectomy, 2 were appendectomy, 2 were re-operation due to intra-abdominal enlarged lymph nodes, 1 was coronary artery bypass grafting (CABG); also, 1 patient required surgery for spinal conditions, 1 for frozen shoulder, 1 for toe amputation (burn injury), 1 for abscess and necrosis in the leg (after drug injection), 1 (a female patient) needed a curettage, and another underwent body-contouring surgery. List of patients requiring additional surgery is depicted in Table 6.

**Table 6:** Of the 26 cases referred for additional surgery, 15 required cholecystectomy leading to a decision to incorporate routine cholecystectomy into the primary surgical intervention

Additional surgical interventions: *n*, 26 (7.22 %)
Cholecystectomy: *n*, 15 (15/61 = 24.6 %)
Appendectomy: *n*, 2 (0.55 %)
Abdominal enlarged lymph nodes: *n*, 2 (0.55 %)
Abscess and necrosis of the leg: *n*, 1 (0.27 %)
Spine surgery: *n*, 1 (0.27 %)
Frozen shoulder: *n*, 1 (0.27 %)
Toe amputation: *n*, 1 (0.27 %)
CABG: *n*, 1 (0.27 %)
Curettage: *n*, 1 (0.27 %)
Abdominoplasty, mammoplasty: *n*, 1 (0.27 %)

Initially, 61 patients did not undergo cholecystectomy on the basis of normal ultrasonographic findings; however, 15 (24.6 %) of these required cholecystectomy within a mean period of 1 year. Therefore, we switched to a routine cholecystectomy after these initial cases. Two patients presented with abdominal pain, food intolerance, and mesenteric enlarged lymph nodes, with poor response to antibiotic and other medical treatments. These were operated; their lymph nodes were removed and examined pathologically. No specific cause could be identified. Leg abscess and necrosis occurred in a patient after analgesic injection which were treated by repeated wound debridement. Toe amputation was performed 6 months after surgery due to a burn injury in a severely diabetic patient who was in complete remission. One patient underwent body-contouring surgery 18 months after DSIT

The mean operative time, including cholecystectomy was 3.9 (range, 3.1 to 8.2) hours. Mean length of hospital stay was 6.9 days (min 4, max 42). Except for the above-listed complications, no other complications commonly seen in bariatric surgery such as parenchymal organ injury/bleeding, marginal ulcer, sleeve leak, and particularly mesenteric herniation occurred. Twenty-nine of 360 patients (8.1 %) were re-admitted for food intolerance and dehydration. Before surgery, 24 patients (6.7 %) had iron deficiency anemia and 87 patients (24.2 %) had vitamin D deficiency, while 22 patients (6.1 %) had iron deficiency anemia and 71 patients (19.7 %) had vitamin D deficiency after surgery.

## Discussion

In this paper, all complications that have occurred during a 2-year period in patients undergoing DSIT have been reported. The data presented involve only those subjects with at least 6 months of follow-up. Our results show a surgical complication rate of 6.1 % and a mortality rate of 0.27 % in our study group. The metabolic outcomes of DSIT have been previously reported [5–7], and the metabolic outcomes in our study will be subjected to future publications. Thus, we have initially intended to report the surgical outcomes for an intervention blamed for surgical complexity. The results presented herein demonstrate a fairly high morbidity rate compared to other bariatric procedures (particularly non-stapled/non-anastomotic procedures) [8, 9]. There was only one mortality within the first month among 360 patients, corresponding to a mortality rate of 0.27 %. This figure is similar to the mortality rates of common bariatric procedures (0.2–0.5 %), which is similar to the mortality rates associated with elective laparoscopic cholecystectomy [10]. The mortality in our series was due to an anastomotic leak (ileo-jejunostomy = third anastomosis). This anatomical site represents the junction between the alimentary and biliopancreatic limbs. Not surprisingly, leaks in this junction are known to be associated with higher mortality rates due to the chemical properties of the biliary and pancreatic juices [11]. The patient who died of the leak was diagnosed on the morning of the first postoperative day and was promptly taken to the operation room (OR). The leak was closed with 3/0 PDS sutures using the routine closure technique; the cavity was washed and drained sufficiently. However, the patient developed omental necrosis 3 days after the revision surgery and did not respond to repeated laparotomies. He died 20 days after the initial procedure.

In total, there were four anastomotic leaks (1 ileo-ileal and 3 jejunoileal). As previously stated, one patient with jejunoileal leak died, and other three patients recovered fully after the revision surgery, all of which involved laparoscopic procedures.

Other surgical complications included bleeding, infections, strictures, and deep vein thrombosis. Most common complications reported after bariatric operations are anastomotic leak, hemorrhage, wound infection, and pulmonary events [12]. This is similar to our findings except for pulmonary events. No major pulmonary events occurred in our patients. All operations in our participants were performed laparoscopically, and all patients underwent a comprehensive checkup program 1 month before surgery, including pulmonary function tests and pulmonary examination. All patients with an underlying pulmonary problem received a 1-month treatment protocol monitored by PEF meter and this routine procedure assisted in the identification of any pulmonary disturbances that may interfere with the outcome.

Two of our patients experienced a bile leak from the duodenal stump, and both were managed conservatively. Another two had bile leakage, one from the cystic duct (misplaced clips) and one from a Luschka channel after difficult simultaneous cholecystectomy, and these were repaired surgically. Initial procedures did not involve routine simultaneous cholecystectomy with DSIT. However, 15 out of initial 61 cases had several problems or developed gallbladder stones, and therefore, we then started to perform routine simultaneous cholecystectomy. Even the leak rates in cholecystectomy performed by the junior members of the surgical team at the final stage of surgery were 0.62 %, which is comparable to those observed in elective cholecystectomy [10]. In total, we had three cholecystectomy-related complications (two leaks, one stricture). Other complications included one case of wound infection and one case of deep vein thrombosis. When these five patients were excluded, the resultant surgery-related technical complication rate was 4.72 % (17/360).

There were two anastomotic strictures, both of which were localized at the duodeno-ileostomy site (second anastomosis). In our routine surgical practice, this anastomosis is performed using a single-layer continuous hand-sewn technique with 3/0 PDS. Vascular supply to the duodenal end of the stomach is solely dependent on the left gastric artery. It is not uncommon to observe a bluish duodenal end while doing this anastomosis. But, stricture requiring stenting occurred in two cases (0.55 %). One male patient totally improved after a single-coated stent application. The other case, a female patient, had an anatomic abnormality whereby the left gastric artery was arising from the hepatic artery. In order to take down the sleeved stomach to the lower abdomen, we routinely sacrifice the right gastric artery, which probably was the dominant artery to the stomach. This might have interfered with the anastomotic blood supply. She re-developed a stricture after the initial stent and developed an anastomotic tear during the application of the second stent. She was taken to the operation room, tear was drained, and a partially covered stent was placed. She did very well after the second stent.

We also had partial sleeve angulation in three cases all of which allowed the advancement of the endoscope for a 1.5-cm diameter length and were treated with endoscopic stent placement. The remaining surgical complications included one wound infection and one deep vein thrombosis. Both were managed conservatively.

We also had non-surgical complications, mainly related to the changes in bowel habits occurring in less than 3 % of the whole patient population. The most frequent complaint was diarrhea followed by constipation. Majority of bowel complaints improved with medical treatment and diet, with lesser requirement for medication and severity of complaints overtime. Patients with food intolerance required hospitalization, and oral or IV supplementation was administered. This problem also tended to improve with time. In total, only two patients (0.55 %) suffered from diarrhea that lasted more than 1 year. The occurrence of nocturnal diarrhea in these two patients points out to a possibility of autonomic neuropathy, rather than steatorrhea [13]. We also believe that itching may also represent a form of neuropathy [14, 15], since no hepatic, renal, or other obvious causes could be identified. Three percent of our patients had neurological complications. These cases were suffering from diabetic neuropathy on the background of long-existing diabetes (mean 12.4 years) and insulin use. Amnesia is a well-documented effect of repeating hypoglycemia, which, in our cases, might be attributable to the consequence of the disease itself [16, 17].

Despite the presence of data indicating improvement in diabetic neuropathy after gastric bypass surgery [18], we observed EMG-confirmed worsening PNP in three cases (0.83 %), all under complete diabetic remission after surgery. Neuropathy after bariatric surgery is an extremely rare complication occurring in less than 0.1 % of cases and is usually associated with severe weight loss, nausea, and vomiting [19, 20]. Osteomalacia and other forms of nutritional deficiencies as well as acute normalization of dysglycemia in some cases have been proposed to play a role in the development of neuropathies after bariatric surgery [21–23]. Our patients experienced worsening of the already existing PNP, rather than new onset PNP. Furthermore, they were within the first 6 months after surgery and had good food tolerance. None of them had nausea or vomiting and were receiving prophylactic multivitamin and mineral supplements. We believe that the acute glycemic regulation might have played a role in our patients. An additional three patients experienced a unilateral foot drop (peroneal palsy) within the first 3 months after surgery that was probably due to sudden weight loss, rather than vitamin or mineral deficiency [24, 25]. They responded well to medical treatment and healed without any sequela. Finally, another three patients experienced a cerebrovascular event, one ischemic, and two hemorrhagic. None of them were fatal. These three patients had had all components of the metabolic syndrome, one had a previous history of MI, and two had carotid stenosis.

In these patients, an important indicator of the safety of this procedure is the requirement for additional surgery in the long term. None of our patients needed a revisional surgery. However, one fourth of the initial 61 cases (15/61, 24.6 %) required a cholecystectomy within 2 years after the initial surgery, despite treatment with oral ursodeoxycholic acid. Two cases presented with pericholecystic abscess. Therefore, we switched to routine cholecystectomy after this initial group of patients. In addition to cholecystectomy, a variety of other surgical interventions were performed. Only two cases underwent laparoscopic exploration due to abdominal pain and enlarged mesenteric lymph nodes. The number of additional surgical procedures performed was 17 in 360 patients (4.72 %). The remaining were two appendectomies, one CABG, one spinal surgery, one surgery for frozen shoulder, one for toe amputation (burn injury), and one for abscess and necrosis in the leg (after drug injection). One patient presented with uterine wall thickening and required curettage. DePaula et al. recently published their paper in 2012 with 39 months of follow-up after DSIT in patients with T2DM and BMI <35 with a mortality rate of 1 % and major morbidity rate of 8.4 % with an early control of postprandial glycemia. This does not seem to compare favorably to published morbidity/mortality rates regarding the sleeve and gastric bypass [26].

It is well established that patients with metabolic syndrome have a predisposition for the development of gallstones and gallstone formation after bariatric surgery [27–29]. Despite the use of routine postoperative ursodeoxycholic acid treatment, we have noted a high incidence of gallstone formation in our initial patient group consisting of 61 subjects, and therefore, we switched to routine cholecystectomy. Apart from gallbladder-related problems, patients undergoing bariatric surgery may develop incisional, ventral, trocar, and internal hernias requiring surgery [30–32]. No herniations were observed in our patients. Finally, one major reason for additional surgery after bariatric or metabolic operation is the reconstructive or body-contouring surgery [33]. In our series, only one female patient underwent abdominoplasty and mammoplasty 18 months after DSIT.

The main limitation of our study group was our average hospital stay which seems almost three times longer than currently published hospital stays in regard to the gastric bypass and sleeve gastrectomy. This increase in hospital stay could lead to increase hospital costs. Although we mentioned the obesity epidemic with reference to disease rates in the USA, the patients included in this study show low BMI. This disparity between the impact of type 2 diabetes in lower BMI patients from Turkey and India versus those from the USA, where bariatric surgery is not readily approved for patients whose BMI is between 25 and 34.9, needs to be emphasized to justify the morbidity/mortality rate accepted for this procedure when compared to that of the natural course of disease in these countries.

The results of this retrospective study clearly demonstrate that DSIT operation can be safely performed in overweight, obese, and morbidly obese type 2 diabetic patients. More objective criteria are certainly necessary for a comparative assessment of different operational techniques and for surgical training to clarify when and who should perform this operation.

In conclusion, after a follow-up of more than 1 year, DSIT has been shown to be a relatively safe surgical alternative for the treatment of T2DM patients with a wide range of BMI. Further studies are warranted to assess the efficacy, patient selection criteria, and cost-effectiveness of the procedure and its effects on reproducible weight loss and resolution or improvement of obesity-related comorbidities especially in long term.
